# Effect of Duration of Dietary Rapeseed and Soybean Oil Feeding on Physical Characteristics, Fatty Acid Profile, and Oxidative Stability of Pig Backfat

**DOI:** 10.3390/ani8110193

**Published:** 2018-10-31

**Authors:** Monika Okrouhlá, Roman Stupka, Jaroslav Čítek, Nicole Lebedová, Kateřina Zadinová

**Affiliations:** Department of Animal Husbandry, Czech University of Life Sciences Prague, Prague, 165 00 Prague Suchdol, Czech Republic; stupka@af.czu.cz (R.S.); citek@af.czu.cz (J.Č.); lebedova@af.czu.cz (N.L.); zadinova@af.czu.cz (K.Z.)

**Keywords:** pig, source of oils, fat quality, malondialdehyde

## Abstract

**Simple Summary:**

The fatty acid profile of pig backfat directly reflects the fatty acid profile of the pig diet. The increased intake of n-3 fatty acids, whose higher content is in vegetable oils, can decrease the risk of heart disease incidence and vascular disorders. The aim of this study was to evaluate the effect of rapeseed and soybean oils and the duration of supplementing the pig diet until slaughter on physical characteristics, fatty acid profile and lipid oxidation of backfat. With the supplementation of oil in the diet for 6 weeks until slaughter, the value of lightness and perforation part of backfat significantly decreased. A dose of 40 g/kg of rapeseed oil in the last 4 weeks before slaughter is sufficient for improving the fatty acid profile without adversely affecting the consistency of the backfat. From the point of view of human health, rapeseed oil is a more suitable source of unsaturated fatty acid than soybean oil in composing the diet for pigs.

**Abstract:**

This study compared the effect of two vegetable oils and their feeding duration on pig backfat quality. The experiment was conducted with 60 DanBred pigs that were fed a diet supplemented (40 g/kg) with rapeseed or soybean oil for 2, 4 or 6 weeks before slaughter at 152 d of age. The supplementation of both vegetable oils in the diets for 6 weeks negatively changed backfat consistency. The pigs fed soybean oil for 4 (17.64%) and 6 weeks (18.52%) before slaughter showed an increase in backfat linoleic acid content (*p* = 0.002), whereas, in contrast to the other groups, rapeseed oil in the diet for 6 weeks (2.38%) increased α-linolenic acid content (*p* = 0.039). The content of PUFAs (*p* = 0.024) and n-6 PUFAs (*p* = 0.003) was increased by adding soybean oil to the diet for 4 and 6 weeks. The ratio of n-6/n-3 PUFAs was decreased (*p* = 0.040) by supplementing rapeseed oil for 4 and 6 weeks. The oil feeding duration decreased (*p* < 0.001) the atherogenic and thrombogenic indices. The lipid oxidative stability of backfat stored for 3 and 5 days increased (*p* < 0.001) in pigs fed dietary vegetable oils for 6 weeks prior to slaughter. In conclusion, the feeding of rapeseed oil for 4 weeks before slaughter is sufficient for improving the FA profile without negative effect on the consistency of the backfat.

## 1. Introduction

Fat addition to diets is important for growing-finishing pigs because of the high-energy value of the diet. In addition, supplementing 1 to 2% fat in the diet will reduce dust in feed and wear of mixing equipment and augurs [1]. For pig diets, there is a wide range of fat sources available. Animal fats, such as beef tallow and lard, are less expensive than vegetable oils, and thus are widely used. However, animal fat has higher saturated fatty acids (SFAs) than vegetable oils.

Consumer preference has increased for products with higher levels of unsaturated fatty acids (UFAs). Because research has confirmed that the increased intake of n-3 fatty acids (FAs) can decrease the risk of heart disease and vascular disorders and can improve the clinical features of some autoimmune and inflammatory disorders [2]. Sakuma and Yamaguchi [3] and Wong et al. [4] recommended that an adequate intake of n-3 polyunsaturated fatty acids (PUFAs) is 1.6 g/day for men and 1.1 g/day for women. The n-6/n-3 PUFA ratio should be less than 5 [5,6]. The most important n-3 FAs are α-linolenic acid (ALA), eicosapentaenoic (EPA), and docosahexaenoic (DHA) acids. These acids can be accreted in animal tissue directly from the feed. The EPA and DHA can be synthesized from precursor α-linolenic acid [7].

The fatty acid profile of pork directly reflects the FA profile of the pig diet [8]. Vegetable oils such as soybean or rapeseed oil contain high levels of UFAs and may lead to healthier products for consumers [9]. On the other hand, the incorporation of PUFAs into the diet is restricted by the resulting decrease in lipid oxidative stability [10], which influences taste, flavour, colour [11], and shelf life [12]. Moreover, feed with a high level of vegetable oil that is rich in UFAs affects the fat consistency (soft and sticky), and thus decreases the quality of meat products [13].

The aim of this study was to evaluate the effect of two sources of fat, and the duration of supplementing the pig diet on physical characteristics, FA profile, and lipid oxidative stability of backfat.

## 2. Materials and Methods 

### 2.1. Animals and Experimental Design

The study was conducted in the pig breeding test station at the Czech University of Life Sciences Prague (Czech Republic). The experiment was approved by the Ethics Committee of the Central Commission for Animal Welfare at the Ministry of Agriculture of the Czech Republic (Prague, Czech Republic) and was carried out in accordance with Directive 2010/63/EU for animal experiments. All procedures described in this study were conducted after obtaining the approval by the Local Ethics Commission, case number 08/2015; the experiment was conducted in CZ21038206.

A total of sixty 70-day-old final hybrid DanBred genotype pigs (*n* = 10; 1:1 gilt and barrow) at an average live weight of 29.2 kg were included in the experiment. The placing and housing of pigs was performed according to methodology described by Stupka et al. [14]. The pigs were housed in pens (two pigs of the same sex per pen) and fed with the complete feed mixture, which contained wheat, barley, soybean meal, and premix. Feed mixture was mixed separately for each pen according to the methodology described by Stupka et al. [14].

The pigs were divided into 6 experimental groups according to the addition of rapeseed or soybean oil and duration of supplying either oil in the pig diet (*n* = 10). The pigs were fed the P1 feed mixture without added oil from the beginning of the experiment until 110 days of age. Then, for 2, 4 or 6 weeks before slaughter the pigs were fed the P3 feed mixture with rapeseed (40 g/kg) or soybean oil (40 g/kg). The groups receiving feed supplemented with oils for 2 or 4 weeks before slaughter were fed the P2 diet until old enough to receive the supplemented P3 diet. The composition and nutritional values of each feed is shown in Table 1 and Table 2. The pigs from all groups were fed ad libitum throughout the experiment.

### 2.2. Carcass Value and Sampling

For the determination of qualitative and quantitative carcass value traits, a carcass analysis of all 60 pigs according to Scheper and Scholz [15] was carried out. For the physical characteristics, fatty acid composition and oxidative stability of backfat, samples from the section between the 1st and 3rd cervical vertebrae were taken. Backfat for fatty acid analysis and oxidative stability was frozen and stored at −80 °C (Jouan HX450S, Trigon-plus, Říčany, Czech Republic) until the analysis.

### 2.3. Physical Characteristics

The colour parameters of lightness (L*; 0 = black, 100 = white), redness (a*; −100 = green, 100 = red), and yellowness (b*; −100 = blue, 100 = yellow) of backfat were measured using a Minolta CM-700d spectrophotometer (Minolta Ltd., Osaka, Japan) 24 h post mortem. The perforation of the upper (between skin and 1st fascia) and lower (under the 1st fascia) part of backfat was detected 24 h post mortem using an Instron Model 3342 (Instron, Norwood, MA, USA) with the injection probe. Each sample was perforated with 6 injections above and 6 under the fascia. The crosshead speed was 200 mm/min with a pressure elongation of 22 mm and sampling rate of 20 mm/s. The maximum perforation (N) was detected. 

### 2.4. Fatty Acid Analysis

The methyl esters of fatty acids after the extraction of total lipids were detected according to the methodology of Folch et al. [16]. Methanolysis was carried out in the presence of KOH and extraction of the acids in the form of methyl esters into heptane. Isolated methyl esters were detected by a flame ionization detector (FID) using the split regime of the chromatography Master GC (Dani Instruments, S.P.A., Milano, Italy) equipped with the Famewax column with polyethylene glycol (Famewax; 30 m × 0.32 mm × 0.25 μm) as the stationary phase. Helium was used as the carrier gas at a constant flow of 5 mL/min, and the split ratio was 1:9. The peaks were identified using Clarity 5.2 and quantified based on known retention times of standards for the Food Industry FAME Mix (Restek Corporation Company, Bellefonte, PA, USA). Atherogenic index (AI) was calculated from monounsaturated fatty acids (MUFA) and PUFA ratio in accordance with the methodology of Chillard et al. [17] as follows: AI = (C12:0 + 4 × C14:0 + C16:0)/(MUFA + PUFA). The thrombogenic index (TI) was calculated according to Ulbright and Southgate [18] as follows: TI = (C14:0 + C16:0 + C18:0)/(0.5 × MUFA + 0.5 × n-6 PUFA + 3 × n-3 PUFA + n-3/n-6 PUFA).

### 2.5. Oxidative Stability

The lipid oxidative stability of backfat was measured immediately after defrosting (0) and the 3^rd^ and 5^th^ days of storage in the fridge at 5 °C. For the extraction, the samples of backfat were homogenized, weighed, and distilled using hydrochloric acid. The results were expressed as thiobarbituric acid reactive substances (TBARS) in mg malondialdehyde (MDA) per kg of backfat. For measuring the absorbance of the colour complex, a Genesys 10vis spectrophotometer (Thermo Fisher Scientific, Madison, Wisconsin, USA) was used at lambda 538 nm against the standard calibration curve 1,1,3,3-tetramethoxypropane.

### 2.6. Statistical Analysis

The experiment was conducted using a 2 × 3 full factorial design. The main effects were the source of oil (rapeseed or soybean oil), the duration of oil feeding (2, 4 or 6 weeks before slaughter) and the interaction between these two factors. The pen was considered as a random effect. The experimental unit was the pen. The data were evaluated by two-way analysis of variance (ANOVA) with the general linear models (GLM) procedure in the SAS 9.4 software (SAS Institute Inc., Cary, NC, USA). The results are presented as the mean and standard error of the mean (SEM). The differences between traits were considered significant when *p* ≤ 0.05.

## 3. Results

### 3.1. Physical Characteristics

The effects of the oil source and duration of supplementation in pig diet on physical characteristics of backfat are shown in Table 3. With the supplementation of oil in the diet for 6 weeks until slaughter, the value of lightness (*p* = 0.018) and perforation of the upper (*p* < 0.001) and lower (*p* < 0.001) part of backfat significantly decreased.

### 3.2. Fatty Acid 

Regarding the FA composition of backfat (Table 4), there was a significant interaction between oil source and duration of feeding for saturated stearic acid *(p* = 0.018), monounsaturated palmitoleic acid (*p* = 0.042), and polyunsaturated linoleic acid (*p* = 0.002) and ALA (*p* = 0.039). Supplementing the diet with rapeseed oil for two weeks before slaughter decreased stearic acid content. Supplementing the diet with soybean oil for six weeks before slaughter decreased palmitoleic acid content. An increase of linoleic acid content was found in the 4- and 6-week soybean oil diet treatments. The ALA content increased after six weeks of feeding with rapeseed oil, contrary to the other groups. In addition, with the increasing length of the feeding period of vegetable oil enriched diets, the content of myristic acid (*p* = 0.007), palmitic acid (*p* < 0.001) and myristoleic acid (*p* = 0.009) decreased, and the content of eicosenoic acid (*p* < 0.001), eicosadienoic acid (*p* < 0.001), eicosatrienoic acid (*p* = 0.032) and EPA (*p* < 0.001) increased. Rapeseed oil in the pig diet increased the percentage of myristoleic acid (*p* = 0.007), oleic acid (*p* < 0.001), eicosenoic acid (*p* = 0.030) and EPA (*p* = 0.023), whereas soybean oil increased the percentage of eicosadienoic acid (*p* = 0.002).

The FA composition of backfat corresponds with sums and ratios of FA and indices (Table 5). Four and six weeks of supplying soybean oil significantly increased the content of PUFAs (*p* = 0.024) and n-6 PUFAs (*p* = 0.003). The ratio of n-6/n-3 PUFAs was decreased (*p* = 0.040) by feeding rapeseed oil for 4 and 6 weeks. The atherogenic index (*p* < 0.001) and thrombogenic index (*p* < 0.001) decreased with the duration of fat feeding. Moreover, lower thrombogenic index (*p* = 0.004) values were detected after using rapeseed oil as the oil source.

### 3.3. Oxidative Stability

Levels of malondialdehyde (MDA) in backfat measured immediately after thawing (day 0) and after storage for three and five days in a refrigerator at 5 °C are shown in Table 6. A statistically significant effect of oil feeding duration was recorded for backfat stored for three and five days (*p* < 0.001). The highest oxidative stability of lipids was ascertained in the backfat of pigs fed both oils for six weeks prior to slaughter.

## 4. Discussion

From the results of the present study, it is evident that the values of backfat perforation significantly decreased after six weeks supplementing the diet with vegetable fats. The negative effect on backfat consistency of feeding with vegetable oils is explained by higher UFA levels in these fat sources [13]. Consistent with our results, Benz et al. [19] stated that increasing feeding duration of soybean oil increased the amount of UFAs leading to softer carcass fat. In addition, Gläser et al. [20] showed that only approximately 30% of the variation in firmness of native backfat could be explained by the fatty acid composition. The amount of superficial fat and therefore thickness of backfat seemed to be more important for firmness of native backfat than its composition. Moreover, there is a strong inverse correlation between the amount of fat and the concentration of PUFAs [21]. Koczanowski et al. [22] found that with the increasing thickness of backfat (from 12 to 16 mm), the concentration of SFAs increased (from 37.7 to 40.4%), while the concentration of PUFAs decreased (from 16.1 to 12.9%).

Rapeseed oil is rich in MUFA and has higher levels of n-3 PUFAs while soybean oil has a high C18:2 content and moderate levels of C18:1 and C18:3. In pigs, dietary FAs are absorbed unchanged from the intestine and incorporated into tissue lipids [23]. Hence, dietary fat influences the FA profile of the adipose tissue [24]. These data correspond with our findings that dietary rapeseed oil increased n-3 FAs and decreased the n-6/n-3 ratio to 4.5:1, whereas soybean oil increased n-6 FAs in backfat. In accordance with our results, Park et al. [25], in a study testing soybean oil as a lipid source for pig diets, showed that the content of PUFA, especially n-3 FAs, in the carcass can be linearly increased depending on the length of the feeding period of a diet supplemented with soybean oil. Similar findings were obtained by Stephenson et al. [26] who stated that increasing the duration of feeding with soybean oil lowered MUFA and increased PUFA concentrations for all fat depots. When pigs are fed diets with n-3 fatty acids, pork and pork products could be recognized as functional foods with new health-promoting properties [27]. This would change the view of pork, which is considered less healthy because the n-6/n-3 PUFA ratio in the meat from pigs fed a commercial feed exceeds the 5:1 recommended by the World Health Organization for meat with health-promoting properties.

Moreover, rapeseed oil is a rich source of oleic acid (54.5% compared to 21.9% in soybean oil in the present study), which has similar effects as the n-3 FAs. It was demonstrated that oleic acid leads to a reduction in cholesterol levels, risk of atherogenesis and high blood pressure [28]. In addition, oleic acid induces beneficial anti-inflammatory effects on autoimmune diseases [29], a protective effect on breast cancer and improvement of immune system function [30]. In the present experiment, we tested two indicators related to human health, the atherogenic and thrombogenic indices. These indices reflect the probability of an increase in pathogenic phenomena, such as atheroma and thrombus formation. The values of the atherogenic and thrombogenic indices were significantly reduced as the rapeseed or soybean oil feeding period increased. In the case of the thrombogenic index, rapeseed oil was more efficient than soybean oil.

Lipid peroxidation is a complex process that is affected by several factors including the degree of saturation. Lipid sources that contain high concentrations of PUFA are highly susceptible to peroxidation [12]. Both soybean oil and backfat of pigs fed soybean oil showed a higher percentage of PUFAs in comparison with rapeseed oil. In this study, however, the malondialdehyde content was not influenced by oil source in fresh backfat nor in backfat stored for three or five days. This was probably because these two vegetable oils are not contrasting fats. A surprising finding was that the feeding of vegetable oils for six weeks decreased malondialdehyde content in backfat stored for five days. This can be explained by the higher oleic acid content in these vegetable oils, especially in rapeseed oil, which can reduce lipid oxidation. In addition, soybean oil used in the feed industry is rich in choline, phospholipids, antioxidants and vitamin E, which prevent oxidation [31]. Similar results were obtained with pork in the study conducted by Alonso et al. [32].

## 5. Conclusions

From the point of view of human health, rapeseed oil is a more suitable source of UFA than soybean oil in composing the diet for pigs. The high content of n-3 FAs in rapeseed oil leads to the reduction of the n-6/n-3 PUFAs ratio to under 5:1 in backfat. An inclusion level of 40 g/kg rapeseed oil in the four weeks before slaughter is sufficient for improving the FA profile without adversely affecting the consistency of the backfat.

## Figures and Tables

**Table 1:** Ingredients and chemical composition of the pig diets.

Indicator	Diets			
	P1	P2	P3 Rapeseed	P3 Soybean
Age of pigs (days)	69–110	111–124/138	111/125/139–152	
Components (g/kg)				
Barley	500	270	620	620
Wheat	313	610	200	200
Soybean meal	150	90	110	110
Premix of vitamins and minerals ^1^	30	30	30	30
Monocalciumphosphate	7	-	-	-
Oil ^2^	-	-	40	40
Chemical composition				
Dry matter (%)	87.6	87.3	88.1	87.9
Crude protein (%)	16.4	14.8	14.5	15.4
Crude fat (%)	1.8	1.8	5.7	5.1
Crude fiber (%)	3.7	1.8	3.8	3.3
Starch (%)	45.4	50.8	44.6	43.8
ME ^3^ (MJ/kg)	13.2	13.6	13.8	13.2
Lysine/ME	0.73	0.60	0.62	0.61
Amino acids (g/kg)				
Lysine	9.64	8.07	8.55	8.33
Methionine	2.95	2.81	2.67	2.65
Threonine	6.24	5.39	5.57	5.53
Tryptophan	2.06	1.74	1.86	1.81
Sulfur amino acids	6.01	5.80	5.38	5.39
Glycine	6.59	6.04	5.74	5.85

^1^ Premix of micro- and macrominerals, essential amino acids, and vitamins −1 kg of premix provided: retinol 400,000 IU, Cholecalciferol 66,000 IU, α-tocopherol 3600 mg, menadione 100 mg, thiamine 60 mg, riboflavin 150 mg, niacin 800 mg, Ca pantothenate 375 mg, vitamin B 6100 mg, vitamin B12 1 mg, choline Cl 15,000 mg, folic acid 15 mg, Fe 3500 mg as FeSO_4_·H_2_O, Zn 3600 mg as ZnO, Mn 3100 mg as MnO, Cu 330 mg as CuSO_4_·5H_2_O, I 175 mg as Ca(IO_3_)_2_, Co 15 mg as 2CoCO_3_·3Co(OH)_2_·H_2_O, Se 13 mg as Na_2_SeO_3_, 6-phytase (EC 3.1.3.26) 25,000 FTU, Ca 220 g, P 20 g, Na 50 g, Mg 10 g, lysine 85 g, methionine 15 g, threonine 15 g; ^2^ rapeseed or soybean oil in diet depending on experimental group; ^3^ calculated metabolizable energy; P1, P2 and P3 = feed mixture. The P3 feed mixture was supplied 2, 4 or 6 weeks before slaughter.

**Table 2:** Fatty acid composition and ratios of oil and diet (LSM).

Fatty Acid (% of Total Fatty Acid)		Oil		Diet	
		Rapeseed	Soybean	Rapeseed	Soybean
Saturated fatty acids					
C14:0	myristic	0.06	0.11	0.06	0.17
C16:0	palmitic	5.59	12.64	9.36	14.57
C17:0	margaric	0.06	0.11	0.07	0.08
C18:0	stearic	1.70	3.85	1.82	3.33
C20:0	arachidic	0.73	0.51	0.59	0.42
C22:0	behenic	0.36	0.34	0.24	0.25
Monounsaturated fatty acids					
C16:1-9*c*	palmitoleic	0.24	0.11	0.20	0.17
C17:1-10*c*	heptadecenoic	0.00	0.06	0.01	0.08
C18:1-9*c*	oleic	54.49	21.88	45.67	20.07
C20:1-11*c*	eicosenoic	1.52	0.34	1.46	0.50
Polyunsaturated fatty acids					
C18:2-9,12*c*	linoleic	23.77	52.61	30.66	52.96
C18:3-9,12,15*c*	α-linolenic	11.25	7.43	9.62	7.24
C20:2-11,14*c*	eicosadienoic	0.06	0.06	0.10	0.08
SFA		8.51	17.57	12.15	18.82
MUFA		56.29	22.39	47.40	20.82
PUFA		35.08	60.09	40.38	60.28
n-6 PUFA		23.77	52.61	30.66	52.96
n-3 PUFA		11.25	7.43	9.62	7.24
n-6/n-3 PUFA		2.11	7.08	3.19	7.31
Atherogenic index		0.06	0.16	0.11	0.19
Thrombogenic index		0.10	0.28	0.16	0.31

LSM = least squares method.

**Table 3:** Physical characteristics of backfat.

	Diet						SEM	Significance		
Items	Rapeseed Oil			Soybean Oil						
	2	4	6	2	4	6		Oil	Time	Oil × Time
Lightness (L*)	77.61	77.11	75.52	77.45	78.13	75.98	0.28	ns	0.018	ns
Redness (a*)	−0.24	−0.62	−0.51	−0.40	−0.51	−0.80	0.06	ns	ns	ns
Yellowness (b*)	7.85	7.37	7.29	7.64	7.32	7.26	0.12	ns	ns	ns
Perforation upper part backfat (N)	61.43	65.56	46.81	67.53	66.95	34.93	7.36	ns	<0.001	ns
Perforation lower part backfat (N)	62.32	66.33	57.43	66.48	72.52	63.44	6.01	ns	<0.001	ns

SEM = standard error of the mean; 2,4,6 = feeding week before slaughter; ns = not significant. L*; 0 = black, 100 = white, a*; −100 = green, 100 = red, b*; −100 = blue, 100 = yellow

**Table 4:** Fatty acid composition of backfat.

Fatty Acid (% of Total Fatty Acid)		Diet						SEM	Significance		
		Rapeseed Oil			Soybean Oil						
		2	4	6	2	4	6		Oil	Time	Oil × Time
Saturated fatty acids											
C 10:0	capric	0.11	0.08	0.08	0.14	0.11	0.09	0.01	ns	ns	ns
C 12:0	lauric	0.10	0.09	0.06	0.14	0.09	0.08	0.01	ns	ns	ns
C 14:0	myristic	1.92	1.76	1.60	1.89	1.88	1.57	0.04	ns	0.007	ns
C 15:0	pentadecanoic	0.05	0.03	0.03	0.03	0.02	0.03	0.00	ns	ns	ns
C 16:0	palmitic	31.20	29.38	26.83	30.46	30.69	26.84	0.34	ns	<0.001	ns
C17:0	margaric	0.34	0.34	0.29	0.31	0.34	0.28	0.01	ns	ns	ns
C18:0	stearic	12.28 ^c^	14.02 ^abc^	14.64 ^ab^	15.01 ^a^	13.17 ^bc^	15.11 ^a^	0.23	ns	ns	0.018
C20:0	arachidic	0.16	0.25	0.34	0.21	0.29	0.31	0.03	ns	ns	ns
C 22:0	behenic	0.00	0.00	0.05	0.04	0.04	0.03	0.01	ns	ns	ns
Monounsaturated fatty acids											
C14:1-9*c*	myristoleic	0.03	0.01	0.00	0.01	0.00	0.00	0.00	0.007	0.009	ns
C16:1-9*c*	palmitoleic	3.27 ^a^	2.31 ^bc^	2.21 ^bc^	2.56 ^b^	2.27 ^bc^	1.98 ^c^	0.07	0.003	<0.001	0.042
C17:1-10*c*	heptadecenoic	0.34	0.28	0.25	0.27	0.24	0.24	0.01	ns	ns	ns
C18:1-9*c*	oleic	35.60	35.32	36.38	31.58	28.43	30.30	0.43	<0.001	ns	ns
C20:1-11*c*	eicosenoic	0.83	1.06	1.46	0.90	0.64	1.17	0.05	0.030	<0.001	ns
Polyunsaturated fatty acids											
C18:2-9,12*c*	linoleic	11.48 ^c^	12.04 ^c^	12.12 ^c^	13.84 ^b^	17.64 ^a^	18.52 ^a^	0.44	<0.001	<0.001	0.002
C18:3-9,12,15*c*	α-linolenic	1.44 ^d^	2.07 ^b^	2.38 ^a^	1.24 ^d^	1.71 ^c^	1.81 ^c^	0.07	<0.001	<0.001	0.039
C20:2-11,14*c*	eicosadienoic	0.43	0.49	0.60	0.55	0.57	0.92	0.03	0.002	<0.001	ns
C20:3-8,11,14*c*	eicosatrienoic	0.06	0.03	0.06	0.06	0.02	0.07	0.01	ns	0.032	ns
C20:4-5,8,11,14*c*	arachidonic	0.10	0.10	0.11	0.13	0.11	0.13	0.01	ns	ns	ns
C20:5-5,8,11,14,17*c*	eicosapentaenoic	0.16	0.21	0.34	0.14	0.14	0.24	0.01	0.023	<0.001	ns
C 22:2	docosadienoic	0.00	0.00	0.01	0.01	0.00	0.01	0.00	ns	ns	ns
C 22:6	docosahexaenoic	0.05	0.03	0.03	0.10	0.02	0.08	0.01	ns	ns	ns

SEM = standard error of the mean; 2, 4, 6 = feeding week before slaughter; ns = not significant; ^a–f^ means in the same row with different superscripts differ significantly.

**Table 5:** Sums and ratios of fatty acids and indexes of backfat.

Fatty Acid (% of Total Fatty Acid)	Diet						SEM	Significance		
	Rapeseed Oil			Soybean Oil						
	2	4	6	2	4	6		Oil	Time	Oil × Time
SFA	46.19	45.97	43.99	48.40	46.67	44.46	0.35	ns	<0.001	ns
MUFA	40.06	38.98	40.36	35.42	31.61	33.77	0.47	<0.001	0.005	ns
PUFA	13.73 ^c^	14.98 ^bc^	15.65 ^bc^	16.08 ^b^	20.22 ^a^	21.77 ^a^	0.50	<0.001	<0.001	0.024
n-6 PUFA	11.59 ^c^	12.14 ^c^	12.23 ^c^	13.97 ^b^	17.74 ^a^	18.65 ^a^	0.45	<0.001	<0.001	0.003
n-3 PUFA	1.65	2.32	2.75	1.49	1.88	2.12	0.08	<0.001	<0.001	ns
n-6/n-3 PUFA	7.18 ^b^	5.25 ^c^	4.45 ^c^	9.72 ^a^	9.46 ^a^	8.83 ^a^	0.29	<0.001	<0.001	0.040
Atherogenic index	0.73	0.68	0.60	0.74	0.75	0.60	0.01	ns	<0.001	ns
Thrombogenic index	1.48	1.39	1.25	1.62	1.51	1.34	0.03	0.004	<0.001	ns

SEM = standard error of the mean; 2, 4, 6 = feeding week before slaughter; ns = not significant; ^a–e^ means in the same row with different superscripts differ significantly.

**Table 6:** Malondialdehyde in backfat (mg MDA/kg)

Backfat Sample	Diet						SEM	Significance		
	Rapeseed Oil			Soybean Oil						
	2	4	6	2	4	6		Oil	Time	Oil × Time
Day 0	0.147	0.266	0.226	0.212	0.164	0.192	0.012	ns	ns	ns
Day 3	0.967	0.936	0.418	0.890	0.808	0.434	0.134	ns	˂0.001	ns
Day 5	1.477	1.524	0.766	1.665	1.701	0.936	0.176	ns	˂0.001	ns

MDA = malondialdehyde; SEM = standard error of the mean; 2, 4, 6 = feeding week before slaughter; ns = not significant.
